# Machine-learning-assisted comparative analysis of rice growth and yield formation in field and plant factory systems

**DOI:** 10.3389/fpls.2026.1875450

**Published:** 2026-06-18

**Authors:** Yujie Yang, Jun Yang, Jie Lu, Yang Tao, Junhua Xie, Tao Zhang, Sen Wang, Qichang Yang

**Affiliations:** 1 Institute of Urban Agriculture, Chinese Academy of Agricultural Sciences, National Agricultural Science & Technology Center, Chengdu, China; 2 Centre for Crop Systems Analysis, Wageningen University and Research, Wageningen, Netherlands; 3 Institute for Advanced Study, Chengdu University, Chengdu, China; 4 College of Horticulture, Henan Agricultural University, Zhengzhou, China

**Keywords:** rice, plant factory, field cultivation, machine learning, phenotypic traits, yield

## Abstract

**Introduction:**

Plant factories provide a controlled platform for rice cultivation and rapid breeding, yet the effects of controlled environments on rice growth and yield formation remain poorly understood relative to field conditions.

**Methods:**

This study systematically compared growth duration, plant architecture, root and leaf traits, biomass accumulation, yield components, and environmental dynamics of three representative rice cultivars grown under field and plant factory conditions. Logistic model fitting was used to characterize plant height growth dynamics, and five machine learning models were further applied to predict plant height and assess the relative importance of growth- and environment-related variables.

**Results:**

The results showed that a plant factory shortened the average growth duration from 138 to 95 days compared with field cultivation and promoted early vegetative development, including stronger tillering, larger total leaf area, longer roots, and greater fresh biomass accumulation. It also increased the total panicles per unit area and the grain number per panicle, whereas seed-setting rate, 1000-grain weight, and final grain yield were not significantly increased. The machine learning models achieved high predictive accuracy for plant height (R^2^>0.9), with growth duration as the dominant predictor in both systems, followed by CO_2_ concentration in the plant factory and cultivar in the field.

**Discussion:**

These findings reveal that controlled-environment cultivation reshapes rice developmental rhythm and vegetative-reproductive allocation, providing a physiological basis for understanding rice plasticity and optimizing plant factory-based rice production.

## 1 Introduction

Rice (*Oryza sativa* L.) is one of the most indispensable staple crops globally, serving as the primary food source for more than half of the world’s population (Hu et al., 2025). With the growing global population, rice demand is expected to increase by 25% from current levels, making stable, high-yield rice production critical to safeguarding global food security (De Clercq and Mahdi, 2025). As China’s most important food crop, rice accounts for 19% of the global planting area and 28% of the global yield, with Southern China a key rice-producing region, where the planting area exceeds 60% of the national total (Jiang et al., 2018; Deng et al., 2019). Despite its vital importance, traditional field rice production faces severe, interconnected challenges threatening its sustainability and productivity: Rapid urbanization and large-scale rural-to-urban migration have triggered severe labor shortages, resulting in soaring cultivation costs and widespread abandonment of paddy fields (Xu et al., 2018); the shift from double-cropping to single-cropping systems, due to labor scarcity and economic pressures, has further reduced annual yields (Zhu et al., 2024); field production is highly vulnerable to climate change, with extreme weather disrupting growth cycles and increasing pest and disease incidence (Itoh et al., 2024); Furthermore, rural-urban demographic shifts, growing competition for land and water resources, and widespread adoption of land-dependent carbon sequestration strategies have together exacerbated shortages of high-quality farmland and irrigation water while threatening to reduce cropland area (Yan et al., 2015; Wang et al., 2019; Gao et al., 2025). Thus, there is an urgent need to develop innovative agricultural systems to overcome these constraints, stabilize production, shorten growth cycles, and accelerate breeding generations to meet growing food demand.

The plant factory, an advanced controlled-environment agricultural system, offers a viable strategy to address the constraints of traditional field production (Blom et al., 2022; He et al., 2024). Unlike open-field conditions, plant factories enable precise, real-time regulation of key environmental factors throughout the entire crop growth period, including light intensity, spectrum, and photoperiod; temperature; relative humidity; CO_2_ concentration; and nutrient supply via soilless culture systems such as hydroponics (Keyvan and Roshandel, 2024; Yuan et al., 2025). This precise environmental control effectively breaks the constraints imposed by natural climate and seasonal variations, providing a novel technical pathway for intensive, sustainable rice production and rapid generation advancement in breeding programs. Previous studies have demonstrated that plant factories, when equipped with LED lighting and optimized nutrient solution management, can significantly improve rice seedling quality, shorten the growth cycle by 40-50% compared to field conditions, and even achieve 5–6 generations of rice per year, a remarkable breakthrough compared to the 1–2 generations achievable through traditional off-season breeding such as southern multiplication (Liu et al., 2024). Furthermore, regulating photoperiod and light quality, combined with carbon-nitrogen coupling management in plant factories, can further promote rice growth and development by optimizing photosynthetic efficiency and nutrient absorption and utilization, thereby supporting the establishment of efficient rice production systems (Liu et al., 2021; Yu et al., 2024).

The intricate interplay among cultivar diversity, variable environmental conditions, and complex field management practices has long posed significant challenges to accurately elucidating multi-factor interaction mechanisms in rice (Zhang et al., 2017, 2024). In recent years, machine learning, a vital branch of artificial intelligence, has demonstrated unique advantages in analyzing complex multivariate relationships (Basso and Liu, 2019; Filippi et al., 2019). Unlike traditional mechanistic models, machine learning can autonomously capture high-dimensional nonlinear patterns from raw data and effectively identify the synergistic and interactive effects among genotype, environment, and management measures. Consequently, it has been widely applied in fields such as crop growth simulation, yield prediction, and the identification of key environmental regulatory factors (Chu and Yu, 2020; van Klompenburg et al., 2020; Dominic et al., 2023). Within plant factory systems, machine learning approaches have been employed to predict the growth of horticultural crops such as tomato (Zhu et al., 2025) and lettuce (Zhang et al., 2025), while relevant research on field crops, including rice, remains relatively scarce. Currently, few studies have used machine learning to quantitatively compare rice growth and development under field and plant-factory cultivation conditions, and the core environmental factors governing rice phenotypic variation and developmental characteristics in these two cultivation systems remain poorly understood. This research gap greatly hinders the advancement of strategies to optimize environmental regulation for rice cultivation in plant factories.

Therefore, to address these knowledge gaps and promote the sustainable development of rice cultivation in plant factories, this study conducted systematic comparative experiments on three representative rice varieties with different genetic backgrounds and growth habits under both field and plant-factory conditions. Five machine learning models, including Decision Tree, Random Forest, Support Vector Machine, Gradient Boosting Decision Tree, and eXtreme Gradient Boosting, were employed to quantify the importance of key environmental and growth-related factors driving rice growth formation. The specific objectives of this study were: (1) to clarify the phenotypic and yield responses of different rice varieties to plant factory conditions, with a focus on differences in vegetative and reproductive growth; (2) to reveal the dynamics of key environmental factors in field and plant factory systems and their respective effects on rice growth and development; (3) to provide a theoretical basis and technical guidance for optimizing environmental regulation strategies and improving the efficiency of rice cultivation in plant factories. By integrating field-to-plant-factory comparisons with machine-learning-based feature attribution, this study aims to reveal how controlled-environment cultivation reshapes rice developmental progression, vegetative-reproductive allocation, and environmentally associated growth regulation.

## 2 Materials and methods

### 2.1 Experimental design

**Figure 1:**
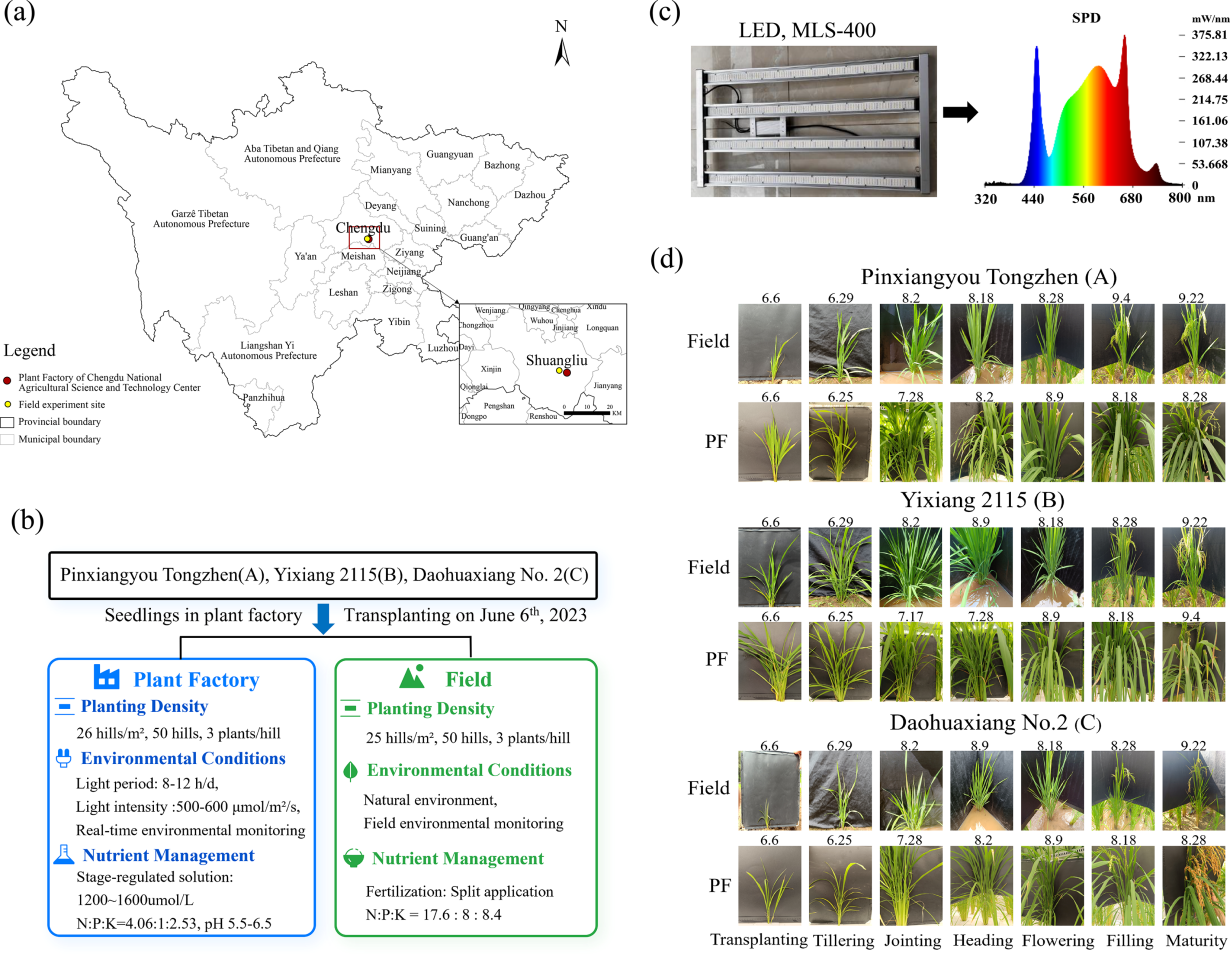
**(a)** Study area; **(b)** Experimental Design; **(c)** MLS-400 LED grow light and its spectral power distribution; **(d)** Different growth stages of three rice cultivars in both field and plant factory conditions with specific dates.

The experiment was conducted in 2023 in Chengdu, Sichuan Province, China, as shown in **Figure 1a**. The plant factory experiment was carried out at the Joint Laboratory of Crop Breeding Plant Factory, Institute of Urban Agriculture, Chinese Academy of Agricultural Sciences/National Chengdu Agricultural Science and Technology Center (104.12°E, 30.41°N). The field experiment was performed at the Rice Experimental Field of Chengdu Institute of Urban Agriculture (104.09°E, 30.42°N). Three rice varieties were used as test materials, namely Pinxiangyou Tongzhen (A), Yixiang 2115 (B), and Daohuaxiang No.2 (C). All seedlings were raised using standardized plug-seedling production in the plant factory and transplanted synchronously into the field and plant factory systems on June 6, when they were uniform.

In the field, a randomized block experimental design was adopted. Each rice cultivar was planted in a 6.0 m² plot, with a uniform planting density of 25 hills/m². Each cultivation system × cultivar combination contained three independent plots as biological replicates. In the plant factory, rice seedlings were cultivated in a hydroponic nutrient solution on double-layer cultivation racks. Each planting board covered an area of 0.62 m × 0.56 m (0.34 m²) with 9 planting holes per board, and the planting density was set at 26 hills/m². Similarly, each treatment combination was replicated three times, with independent planting boards serving as biological replicates. Cultivation management, environmental conditions, and nutrient practices for field and plant factory systems are illustrated in **Figure 1b**. The plant factory was equipped with MLS-400 LED grow lights, whose spectral power distribution (SPD) exhibited a dominant blue peak at 450 nm, a broad green-to-red band centered at 620 nm, a secondary red peak at 660 nm, and a small far-red component around 730 nm, as shown in **Figure 1c**. The photosynthetic photon flux density (PPFD) was maintained at a constant level across the plant canopy throughout the experiment. Key growth stages, including tillering, jointing, heading, flowering, filling, and maturity, were systematically recorded throughout the growth period, as presented in **Figure 1d**. Daily maximum/minimum/average temperature (T_max_, T_min_, T_avg_), relative humidity (H_max_, H_min_, H_avg_), and CO_2_ concentration (CO_2max_, CO_2min_, CO_2avg_) were continuously monitored in both systems.

### 2.2 Plant height fitting and growth stage recording

After transplantation, six uniform plants per variety were randomly selected for periodic plant height measurements at scheduled time points: June 6, 9, 17, 25, and 29; July 4, 14, 17, and 28; August 2, 9, 18, and 28; and September 4, 13, and 22. The critical-point-based Logistic model (Hsieh et al., 2026) was applied to characterize the growth dynamics of three rice cultivars under field and plant factory conditions. Concurrently, the timing and duration of key growth stages including tillering, jointing, heading, flowering, filling, and maturity were recorded for each variety under both cultivation systems, with each stage defined based on morphological characteristics (Counce et al., 2000): tillering was recorded when the first tiller emerged; jointing was defined as when the node of the uppermost internode clearly elongated; heading was scored when more than 50% of panicles completely emerged from the sheath; flowering was identified when more than 50% of panicles exhibited exserted anthers; filling was determined when endosperm accumulation became visible in the grains; and maturity was defined when grains reached maximum dry weight and leaf senescence occurred. The duration of each stage and the total growth period were then calculated, and differences in rice growth progress and population dynamics between field and plant factory conditions were statistically analyzed and compared.

### 2.3 Phenotypic and yield trait measurements

At physiological maturity, six uniform plants were randomly selected from each treatment to quantify phenotypic traits, biomass accumulation, and yield components. Plant height was measured from the base of the plant to the tip of the main panicle, while stem length was recorded from the stem base to the panicle neck and panicle length from the neck to the panicle tip; the length and diameter of the 1st to 4th internodes (counted basipetally) were determined using a Vernier caliper. Root length was measured as the distance from the stem base to the tip of the longest root. Leaf traits, including total leaf area per plant, flag leaf area, and the area of the second and third top leaves, were accurately determined using LI-3100C desktop leaf area meter. For biomass analysis, leaves, roots, stems, and panicles were harvested, separated, and weighed to record fresh weights. All samples were then oven-dried at 80 °C to a constant weight for dry weight determination. Yield components, including total panicles per unit area, grain number per panicle, total spikelets per unit area, seed-setting rate, 1000-grain weight, and actual grain yield, were quantitatively measured and calculated in accordance with national standard protocols for rice yield surveys, and grain yield was converted to a per-hectare basis.

### 2.4 Machine learning modeling and parameter settings

Five machine learning models were applied to predict rice plant height. The models used include Decision Tree, Random Forest, Support Vector Machine, Gradient Boosting Decision Tree, and XGBoost. All models were implemented using R packages including “rpart”, “randomForest”, “e1071”, “gbm”, and “xgboost” (Bischl et al., 2016). The dataset was divided into a training set (70%) and a test set (30%), with a fixed random seed of 123 to ensure reproducibility. For each model, hyperparameters were predefined to ensure stability and comparability, and five-fold cross validation was adopted for model evaluation. Specific parameters were set as follows: for Decision Tree, the ANOVA method was used with a maximum tree depth of 5; for Random Forest, the number of trees was set to 500 with three randomly selected variables at each split, and feature importance was recorded; for Support Vector Machine, features were standardized and centered, the radial basis function kernel was employed, the penalty parameter C was set to 1, and gamma was set to 0.2; for Gradient Boosting Decision Tree, the Gaussian distribution was used with 500 trees, a maximum interaction depth of 3, a learning rate of 0.1, and a subsample ratio of 0.7; for XGBoost, the DMatrix data structure was used with the reg:squarederror objective, a learning rate of 0.1, a maximum tree depth of 3, a row subsample ratio of 0.7, a column subsample ratio of 0.8, with a maximum of 500 iterations and early stopping after 50 rounds.

### 2.5 Model validation and feature importance analysis

An enhanced evaluation framework was constructed, and tailored prediction strategies were adopted for each model. All experimental samples were randomly divided into a 70% training set for model fitting and a 30% independent test set for validation of predictive performance. Five quantitative indicators were used to comprehensively evaluate model accuracy and generalization, including Root Mean Square Error (RMSE), Coefficient of Determination (R²), Mean Absolute Error (MAE), Mean Absolute Percentage Error (MAPE), and Nash-Sutcliffe Efficiency (NSE). Furthermore, feature importance analysis was performed using model-specific algorithms: built-in variable importance was extracted for DT, %IncMSE was adopted for RF, permutation importance with 20 repetitions was used for SVM, relative influence (rel.inf) was calculated for GBDT, and feature gain was used as the indicator for XGBoost. The relative importance percentage for each feature was computed for each model individually. Subsequently, the mean, median, and standard deviation of relative importance across the five models were calculated to obtain the average feature importance.

### 2.6 Statistical analysis

All experimental data were processed using Excel 2019 and R 4.2.2. An independent-samples t-test was used to determine significant differences between the two cultivation systems at P < 0.05, P < 0.01 and P < 0.0001.

## 3 Results

### 3.1 Dynamic changes of environmental factors

**Figure 2:**
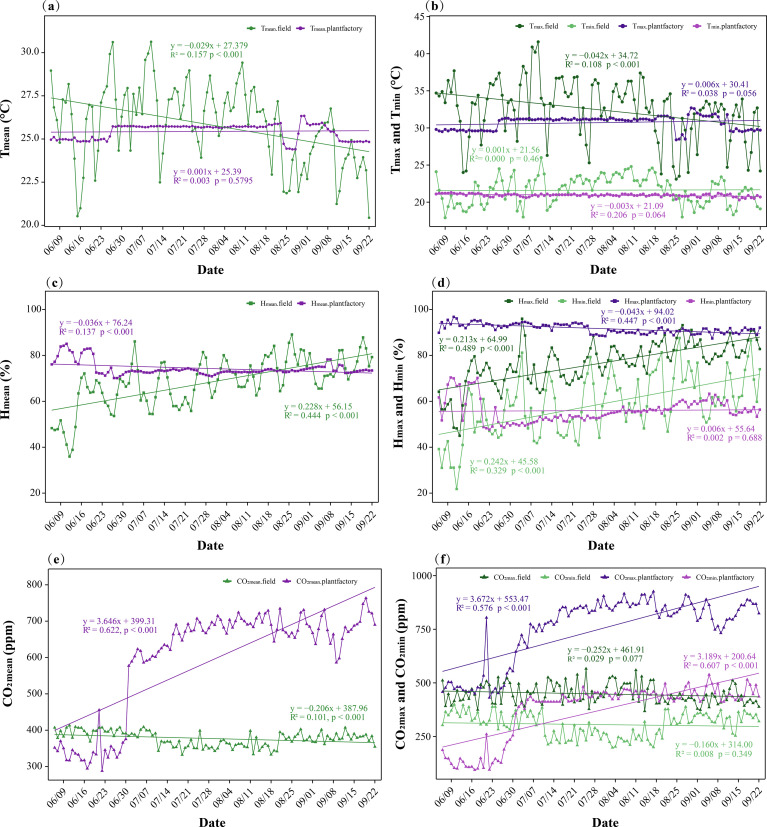
Dynamic changes of environmental factors during the rice growth period under field and plant factory cultivation conditions. **(a)** Mean temperature (T_mean_​); **(b)** Maximum and minimum temperature (T_max_​, T_min_​); **(c)** Mean relative humidity (H_mean_​); **(d)** Maximum and minimum relative humidity (H_max_​, H_min_​); **(e)** Mean CO_2_ concentration (CO_2mean_​); **(f)** Maximum and minimum CO_2_ concentration (CO_2max_​, CO_2min_​). Green lines represent the field environment, while purple lines represent the plant factory environment. Linear regression lines, equations, coefficients of determination (R^2^), and significance levels (P) are shown.

From early June to late September, dynamic changes and linear fitting trends of key environmental factors, including CO_2_ concentration, relative humidity, and temperature, between the field and plant factory, as shown in **Figure 2**. Temperature in the field showed a highly significant linear decrease with growth progression, with daily maximum and mean temperatures declining significantly (P<0.001), whereas no significant trend was observed for daily minimum temperature (P>0.05). In contrast, temperatures in the plant factory remained highly stable throughout the growth period, with no significant linear changes in maximum, mean, or minimum temperature (P>0.05), and the fitted curve was nearly flat, indicating a constant and suitable thermal environment. Relative humidity in the field increased significantly over the growing period, as daily maximum, mean, and minimum humidity all showed significant upward trends (P<0.001). By contrast, in the plant factory, daily mean and maximum humidity decreased significantly (P<0.001), while minimum humidity remained stable with no significant linear change (P>0.05). The mean CO_2_ concentration in the plant factory increased significantly over time, with a slope of 3.189 and a coefficient of determination (R²) of 0.607 (P < 0.001). Daily maximum and minimum CO_2_ concentrations also increased sharply (P<0.001), and all CO_2_ levels in the plant factory were notably higher than those in the field. This elevation was primarily attributed to passive gas accumulation from crop respiration within the sealed cultivation space, rather than to manual environmental regulation. By contrast, the mean CO_2_ concentration in the field showed a significant decreasing trend (P<0.05), while no significant linear changes were observed for daily maximum and minimum CO_2_ concentrations (P>0.05). In summary, the plant factory provided a highly controllable and controlled environment with stable temperature, gradually increasing humidity, and continuously rising CO_2_ concentration, supporting the rapid growth and development of rice.

### 3.2 Plant height fitting and stem traits

Throughout the growth period, the dynamic changes in plant height among the three rice varieties differed between the plant factory and field conditions. In the plant factory, plant height increased rapidly during the early growth stage (0-60d) and gradually stabilized in the later stage. In contrast, field-grown rice exhibited a steady and continuous increase in plant height, ultimately surpassing plant factory crops across all three varieties during the late growth stage, as shown in **Figurse 3a–c** and **Table 1**. Plant height data for all treatments were well fitted by the Logistic model, with all R^2^ values exceeding 0.97, demonstrating a typical sigmoidal growth pattern in rice height. Rice grown in the field had a higher theoretical maximum plant height ranging from 125.55 to 156.09 cm. The peak growth rate occurred 40.11 to 44.72 days after transplanting, and the rapid growth period lasted 95.88 to 168.54 days. In comparison, rice in the plant factory reached a lower theoretical maximum height of 90.56 to 107.93 cm, and its growth rhythm was distinctly advanced, with the peak growth stage appearing much earlier at 10.85 to 25.83 days.

**Figure 3:**
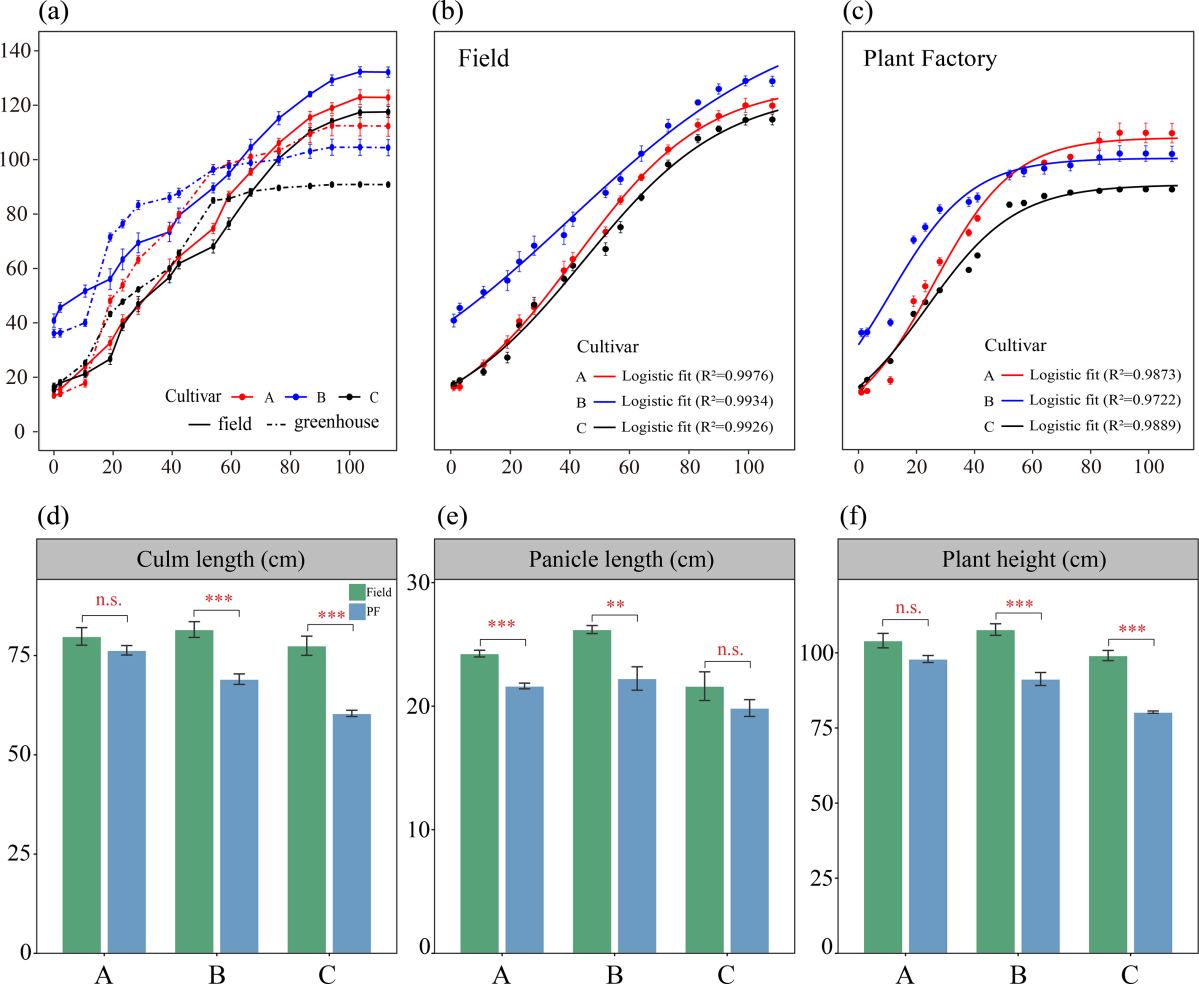
Plant height dynamics and mature traits of rice under field and plant factory conditions. **(a)** Plant height growth curves across both cultivation systems. **(b, c)** Logistic model fitting of plant height growth for rice grown in the field and plant factory, respectively. **(d-f)** Comparisons of culm length, panicle length, and final plant height at maturity. Error bars represent standard error. Values were compared via t-test; **P<0.01, ***P<0.001, n.s. means no significant difference. A: Pinxiangyou Tongzhen, B: Yixiang 2115, C: Daohuaxiang No.2.

**Table 1:** Key growth parameters of rice plant height fitted by the logistic model.

	Cultivar	K (cm)	R^2^	T_max_ (day)	R_max_ (cm/day)	T_strat_ (day)	T_end_ (day)	RGD (day)
Field	A	127.78	0.9976	42.04	1.464	-5.9	89.98	95.88
	B	156.09	0.9934	40.11	1.017	-44.16	124.38	168.54
	C	125.55	0.9926	44.72	1.323	-7.4	96.83	104.23
Plant Factory	A	107.93	0.9873	25.83	1.948	-4.6	56.27	60.88
	B	100.32	0.9722	10.85	1.793	-19.88	41.59	61.46
	C	90.56	0.9889	23.51	1.478	-10.15	57.16	67.31

Note: K, theoretical maximum plant height; R^2^, coefficient of determination; T_max_​, day of maximum growth rate; R_max_​, maximum growth rate; T_start_​, start day of rapid growth; T_end_​, end day of rapid growth; RGD, rapid growth period. A: Pinxiangyou Tongzhen, B: Yixiang 2115, C: Daohuaxiang No.2.

**Figure 4:**
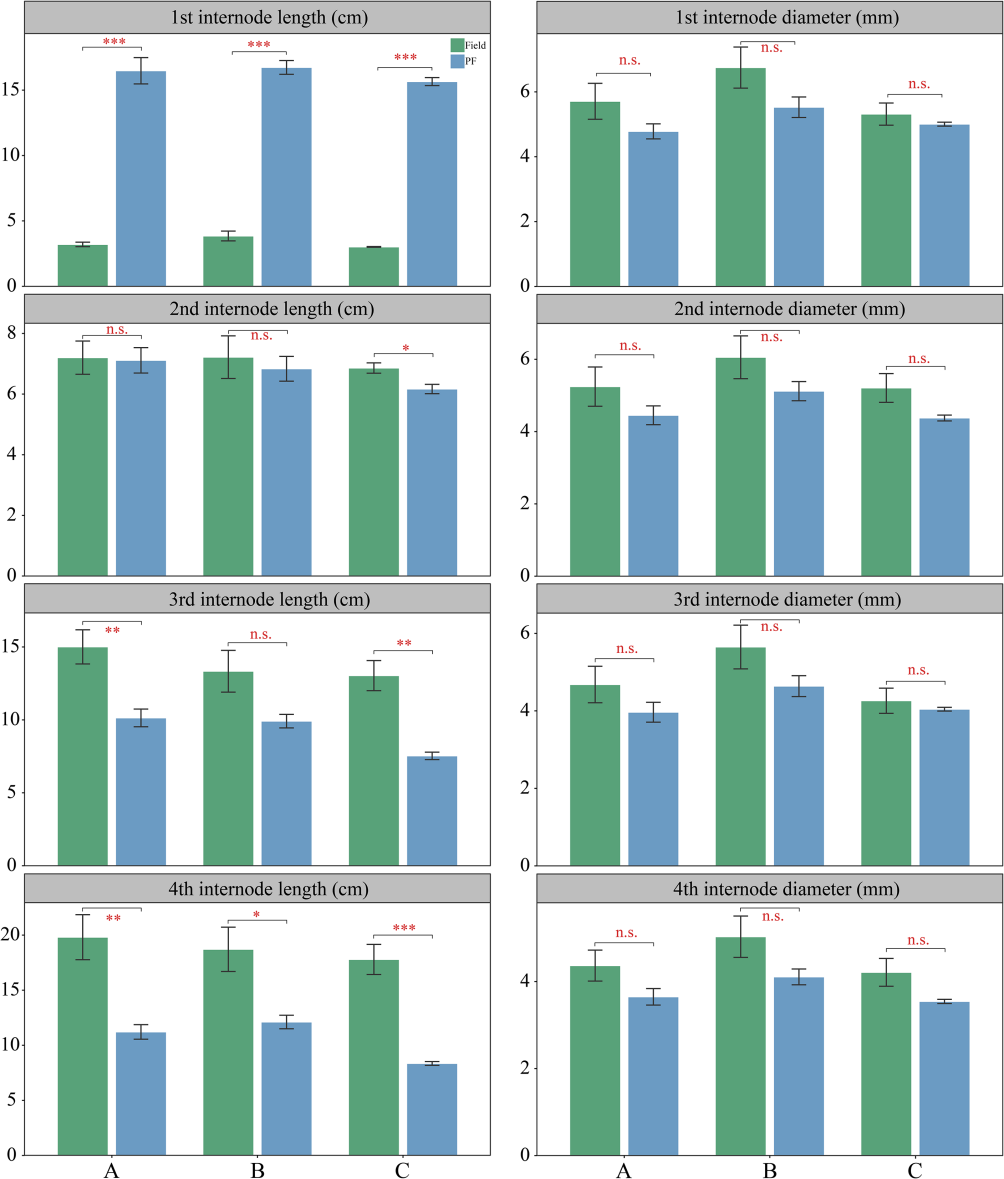
Internode morphological traits of rice at different positions under field and plant factory conditions. Error bars represent standard error. Values were compared via t-test; *P<0.05, **P<0.01, ***P<0.001, n.s. means no significant difference. A: Pinxiangyou Tongzhen, B: Yixiang 2115, C: Daohuaxiang No.2.

Analysis of maturity traits revealed clear differences in responses to culm length, panicle length, and plant height between the two cultivation environments, as presented in **Figures 3b–d**, with vegetative and reproductive organs exhibiting distinct environmental sensitivity. Specifically, panicle length of Variety A was significantly higher in the field than in the plant factory (P<0.05), whereas stem length and plant height showed no significant difference (P>0.05). Variety B exhibited significant differences in panicle length, stem length, and plant height between the two environments (P<0.05). For Variety C, stem length and plant height were significantly higher in the field (P<0.05), while panicle length showed no significant difference (P>0.05). The length and diameter of the 1st to 4th internodes of the three rice varieties showed significant internode-specific responses under plant factory and field cultivation, with no consistent pattern among different internodes, as shown in **Figure 4**. For internode length, the 1st and 4th internodes of all three varieties were significantly longer in the plant factory than in the field (P<0.05). The 2nd internode length showed no significant difference between the two cultivation systems in varieties A and B (P>0.05). The 3rd internode length did not differ significantly in variety B (P>0.05) but differed significantly in varieties A and C (P<0.05). Notably, the lengths of the 1st to 4th internodes in variety C were all significantly different between the two cultivation modes (P<0.05). For internode diameter radial growth, the diameter of all internodes remained generally stable and showed no significant changes in all three varieties under either plant factory or field conditions (P>0.05).

### 3.3 Leaf and root traits

**Figure 5:**
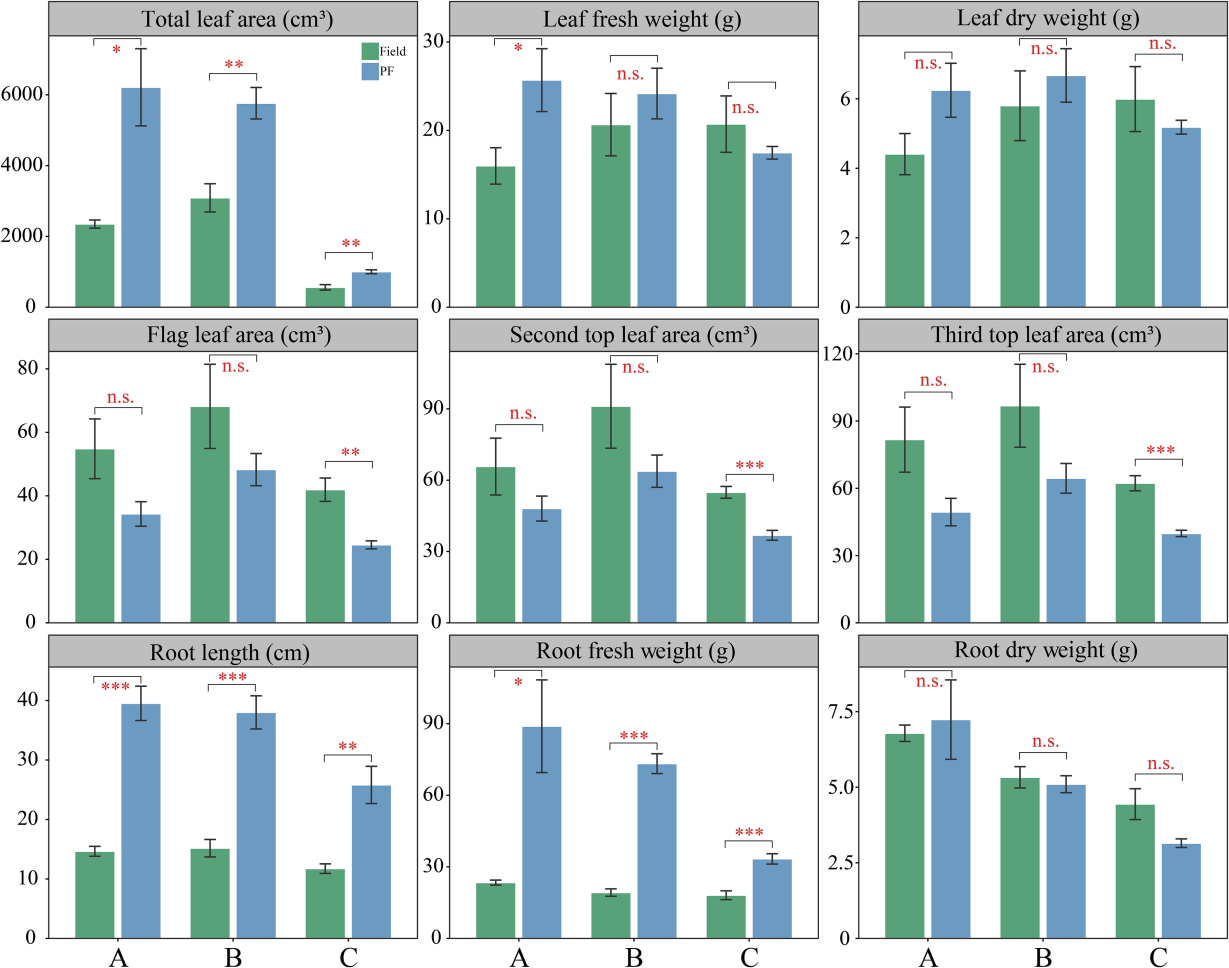
Leaf and root traits of three rice cultivars under field and plant factory conditions. Error bars represent standard error. Values were compared via t-test; *P<0.05, **P<0.01, ***P<0.001, n.s. means no significant difference. A: Pinxiangyou Tongzhen, B: Yixiang 2115, C: Daohuaxiang No.2.

Leaf and root traits showed obvious organ−specific and cultivar−specific responses between field and plant factory cultivation systems, as shown in **Figure 5**. Compared with the field, the plant factory significantly increased total leaf area in all three cultivars, with significant differences in cultivar A (P<0.05) and highly significant differences in cultivars B and C (P<0.01), indicating that the controlled environment strongly promotes leaf expansion during vegetative growth. However, as the main photosynthetic organ, flag leaf area did not differ significantly between the two systems for cultivars A and B; only for cultivar C was flag leaf area significantly larger in the field (P<0.01). For the second and third top leaves, no significant differences were observed in cultivars A and B, whereas cultivar C showed significantly larger values under field conditions (P<0.001), revealing strong cultivar-specific responses. Overall, although the plant factory greatly increases total leaf area, it has little effect on the area of key functional leaves. The hydroponic environment in the plant factory markedly promoted root elongation and fresh biomass accumulation. Root length and root fresh weight were significantly higher in the plant factory for all cultivars, with root length differing significantly (P<0.01) and root fresh weight showing similar significant differences (P<0.05). Nevertheless, root dry weight did not increase correspondingly. No significant differences were detected in root dry weight for any of the three cultivars between the two cultivation systems (P>0.05). These results suggest that the plant factory mainly enhances water absorption and fresh matter accumulation in roots, with limited promotion of dry matter partitioning, and the response varies greatly among cultivars.

### 3.4 Yield components

**Figure 6:**
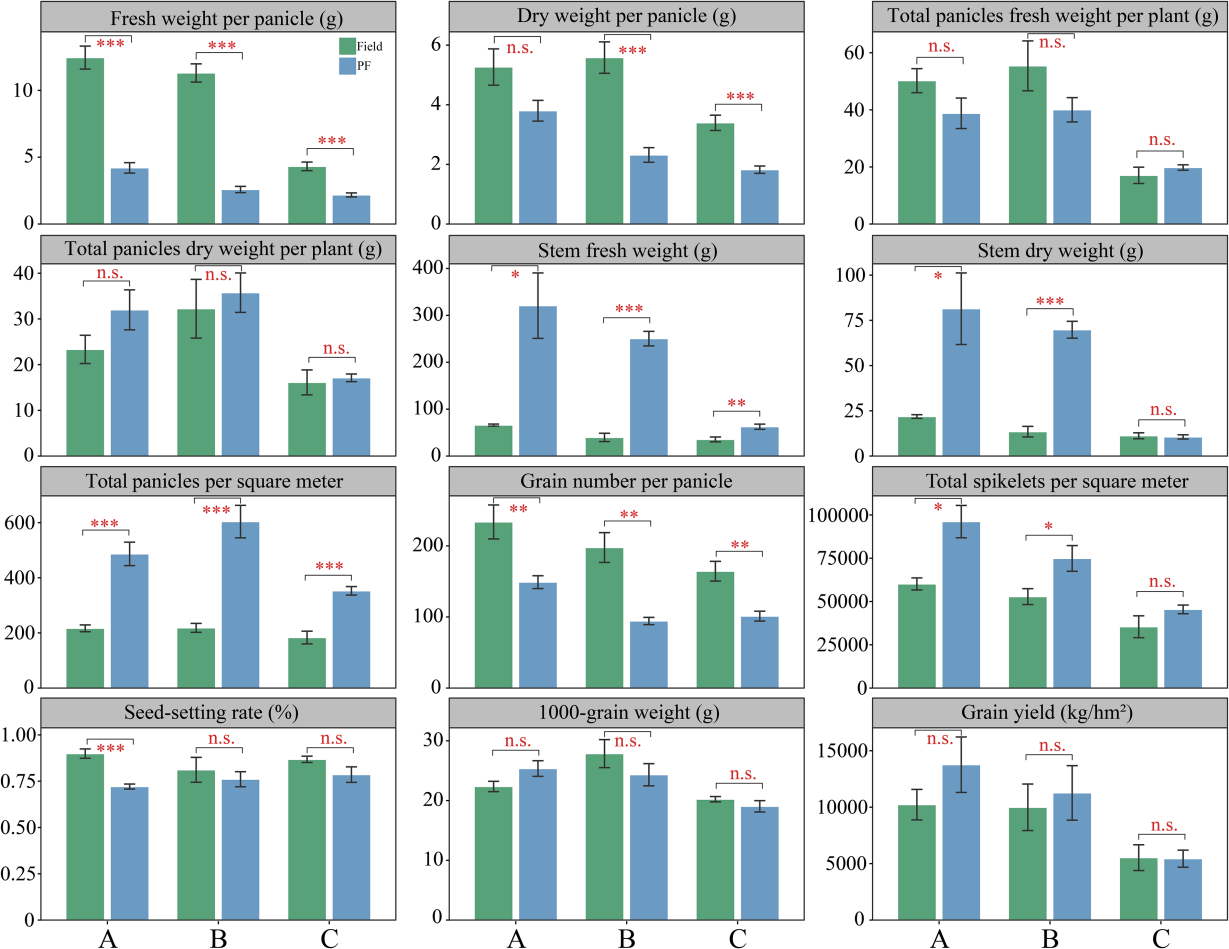
Biomass and yield components of three rice cultivars under field and plant factory conditions. Error bars represent standard error. Values were compared via t-test; *P<0.05, **P<0.01, ***P<0.001, n.s. means no significant difference. A: Pinxiangyou Tongzhen, B: Yixiang 2115, C: Daohuaxiang No.2.

Plant factory and field cultivation exerted distinct environmental regulation on rice yield components as well as stem and panicle biomass, and the differential responses displayed obvious organ−specific and trait−specific patterns, as illustrated in **Figure 6**. In terms of biomass accumulation, plant factory cultivation significantly increased fresh weight per panicle and stem fresh weight (P<0.01), which was consistent with the expansion of sink capacity and the trend of stem elongation. In comparison, no significant differences were observed in dry weight per panicle and panicle biomass per plant between the two cultivation systems (P>0.05). These results indicated that plant factory cultivation mainly promoted fresh biomass and water accumulation in vegetative organs, whereas its promotion of effective dry matter accumulation related to grain filling was limited. This physiological imbalance ultimately kept panicle dry matter stable and resulted in non−significant yield variation between plant factory and field conditions (P>0.05). In terms of yield components, the plant factory significantly increased total panicles per square meter and grain number per panicle in three rice cultivars(P<0.01). Plant factory conditions significantly increased total spikelets per square meter in cultivars A and B (P<0.05), but not in cultivar C (P>0.05). Nevertheless, seed-setting rate and 1000−grain weight remained steady and did not increase in tandem with the enlarged sink capacity (P>0.05), resulting in no significant difference in final grain yield between the two cultivation environments (P>0.05). It was concluded that the superior sink capacity established under plant factory conditions could not be effectively translated into a yield advantage.

### 3.5 Model performance and feature importance analysis

**Figure 7:**
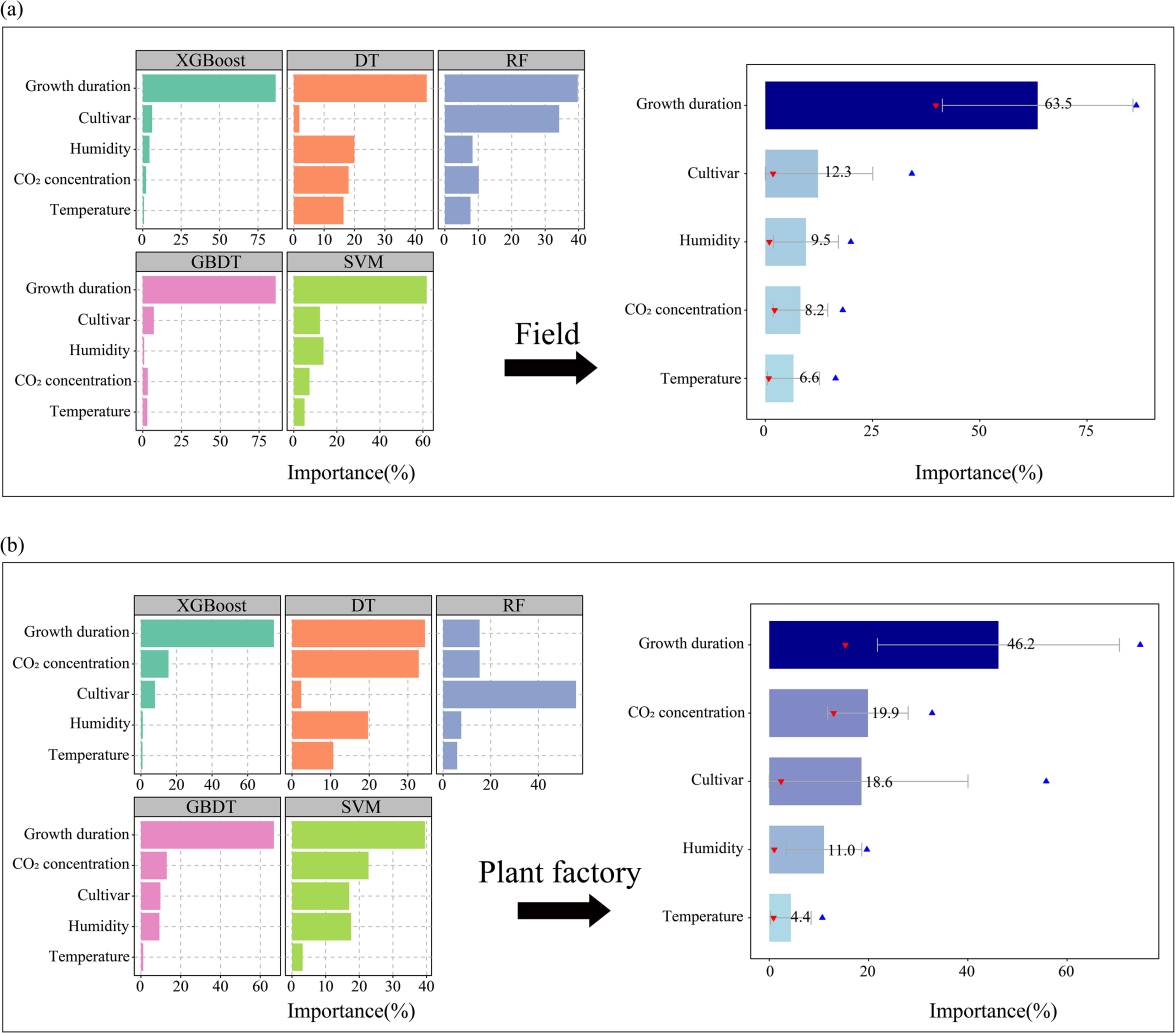
Importance analysis of driving factors for rice phenotypic traits based on machine learning models under field and plant factory cultivation conditions. **(a)** Field cultivation; **(b)** Plant factory cultivation. The left panels show the feature importance of each factor derived from five machine learning models (XGBoost, DT, RF, GBDT, SVM). The right panels present the average importance of each factor across the five models, with error bars indicating the standard deviation.

**Table 2** shows that all machine learning models achieved high prediction accuracy for rice plant height across both cultivation systems, with R² values exceeding 0.91. Under field conditions, XGBoost performed best, with the lowest RMSE and highest R², NSE, and prediction stability. GBDT, SVM, and RF also showed high accuracy (R²>0.97), whereas DT had relatively lower precision. Under plant factory conditions, XGBoost and GBDT performed equally well and exhibited the greatest robustness. SVM and RF maintained strong predictive performance, while DT remained the least accurate model. Feature importance analysis, as shown in **Figure 7**, revealed strong environmental specificity in the contributions of factors. In the field, growth period was the dominant driver, with an average importance of 63.5%, followed by cultivar, humidity, CO_2_ concentration, and temperature. In the plant factory, the growth period remained the primary factor, but its relative importance decreased to 46.2%. Notably, CO_2_ concentration became the second most important factor at 19.9%, followed by cultivar and humidity; temperature remained the least important in both systems. These results confirm that the growth period acts as the genetically determined core driver, while CO_2_ concentration becomes a key environmental regulator in the plant factory.

**Table 2:** Prediction performance of five machine learning models for rice plant height.

	Model	RMSE	R^2^	MAE	MAPE	NSE
Field	XGBoost	5.395	0.977	4.265	9.38	0.977
	GBDT	5.646	0.975	4.434	9.62	0.975
	SVM	5.93	0.972	4.66	10.91	0.972
	RF	6.107	0.971	4.623	10.66	0.97
	DT	10.827	0.913	8.6	16.27	0.907
Plant Factory	GBDT	4.771	0.976	3.154	5.91	0.975
	XGBoost	4.728	0.976	3.011	4.86	0.976
	SVM	5.108	0.974	3.54	7.2	0.971
	RF	5.291	0.972	3.613	7.24	0.969
	DT	8.573	0.927	6.343	15.06	0.92

RMSE, Root Mean Square Error; R², coefficient of determination; MAE, Mean Absolute Error; MAPE, Mean Absolute Percentage Error; NSE, Nash-Sutcliffe Efficiency.

### 3.6 Population dynamics and growth duration

**Figure 8:**
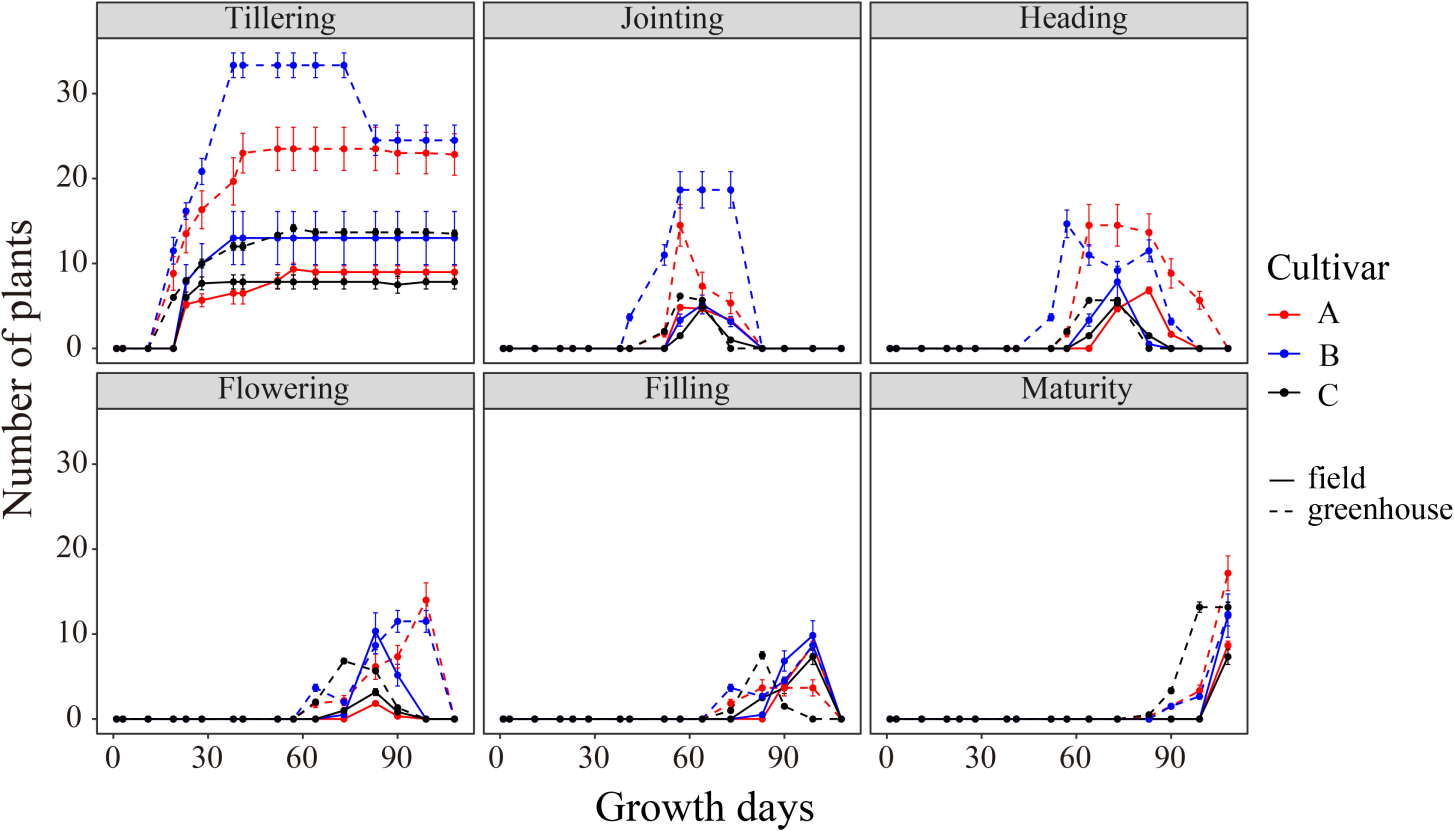
Population dynamics of rice at different growth stages under field and plant factory conditions. Error bars represent standard error. A: Pinxiangyou Tongzhen, B: Yixiang 2115, C: Daohuaxiang No.2.

The dynamics of plant number across key growth stages differed between field and plant factory conditions for all three rice varieties, as shown in **Figure 8**. During the tillering stage, plant numbers increased rapidly, with plant factory-grown plants showing consistently higher counts than those in the field. Variety B exhibited the most vigorous tillering, reaching a peak plant number above 30 in the plant factory, while varieties A and C peaked at approximately 25 and 14, respectively. In contrast, field-grown plants reached lower tillering peaks, with variety C showing the smallest population size. As growth progressed to the jointing and heading stages, plant numbers gradually declined in both environments. Plant factory-grown plants maintained higher counts than field-grown plants during these stages, particularly in variety B, which retained a larger population size. Differences between the two environments diminished in the flowering and grain-filling stages, with plant numbers stabilizing and converging across treatments. At maturity, plant numbers were comparable between field and plant factory conditions for all varieties, although plant factory-grown plants tended to maintain slightly higher counts.

**Figure 9:**
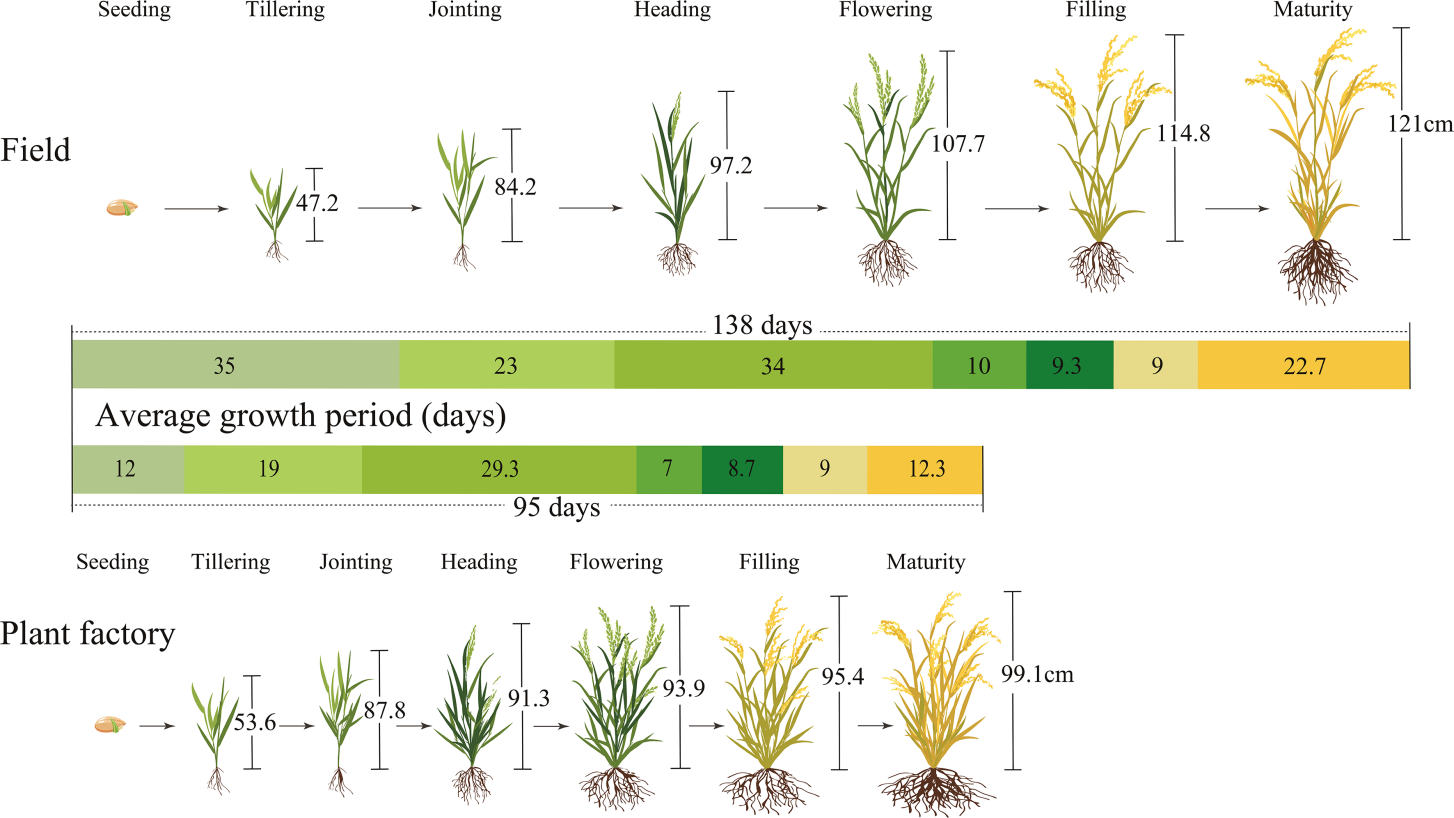
Key stages (seeding, tillering, jointing, heading, flowering, filling, maturity) for rice cultivated in the field and plant factory. The average total growth period was 138 days in the field and 95 days in the plant factory.

As shown in **Figure 9**, the average total growth period was 138 days in the field but only 95 days in the plant factory, indicating that precise environmental regulation in the plant factory significantly shortened the rice growth cycle. Rice grown in the plant factory showed faster early growth and more active tillering, together with greater total leaf area, longer roots, higher root fresh weight, and increased panicle number per unit area. In contrast, field-grown rice exhibited a longer developmental period and greater plant height and panicle length at the later growth stage across several cultivars. Although plant factory cultivation enhanced several vegetative and sink-related traits, including tillering capacity and panicle number, final grain yield did not increase in parallel with field cultivation. Together, these results indicate that plant factory cultivation primarily accelerated early developmental progression and enhanced selected vegetative and sink-related traits, whereas field cultivation enabled a longer growth period and stronger late-stage elongation of plant and panicle organs.

## 4 Discussion

This study systematically compared the growth dynamics, phenotypic traits, environmental variations and growth-driving factors of three rice cultivars grown under field and plant factory conditions. The plant factory markedly shortened the whole growth cycle from 138 days to 95 days and substantially stimulated vegetative growth, including plant height, root development, stem expansion and lower leaf growth (**Figure 9**). By comparison, rice grown in fluctuating field environments exhibited relatively slower vegetative growth but produced taller plants and longer panicles with more robust reproductive development. Logistic model fitting further confirmed that field cultivation sustained longer vigorous growth, which supported steady biomass accumulation and contributed to greater final plant height. In contrast, the artificially regulated plant factory accelerated the instantaneous growth rate and advanced the occurrence of peak growth, yet the compressed rapid growth stage restricted the ultimate growth potential (**Table 1**). Integrated analyses of internode morphology (**Figure 4**) and biomass partitioning (**Figure 6**) demonstrated that the plant factory primarily facilitated dry matter accumulation in vegetative tissues. Nevertheless, it failed to effectively promote dry matter translocation toward grains, resulting in inferior reproductive dry matter allocation relative to field-grown rice. From the perspective of source-sink relationships, source activity (photosynthetic activity) and leaf size (photosynthetic area and its duration) determine the biomass production capacity of the crop source, whereas post-anthesis grain growth, which utilizes existing assimilates, constitutes the sink process (Shi et al., 2017; Burgess et al., 2022). The synergistic effect of source capacity and sink growth jointly determines rice yield (Zhang et al., 2021); therefore, better coordination of source supply and sink demand is often proposed as a feasible approach to improve rice yield (Zhou et al., 2023, 2024). One of the major bottlenecks to improving rice biomass accumulation and grain yield is enhancing photosynthetic capacity. Nevertheless, converting elevated photosynthetic efficiency and photoassimilate production into superior biomass and harvestable yield relies largely on sufficient sink size and efficient translocation of carbohydrates to reproductive sink tissues, particularly developing grains (Dingkuhn et al., 2020).

Moreover, as C3 plants, rice genotypes with larger sink size might benefit from higher carbon fixation under rising CO_2_ (Ziska et al., 2012). Significant genotypic differences in yield performance and yield-component traits were observed among the three rice cultivars in our study. Therefore, genotypes with greater grain sink capacity were capable of fixing more carbon and allocating assimilates rationally, thereby achieving higher yield potential (Anwar et al., 2022). In the context of plant factory cultivation, although controlled environments can greatly promote vegetative growth and structural sink formation, coordinated improvement in reproductive grain sink strength remains the key to breaking yield constraints. For the yield formation in **Figure 6**, the plant factory significantly increased total panicles per unit area and grain number per panicle, thereby enlarging sink capacity (Ueda et al., 2025). Grain-filling duration is a crucial phase that determines rice grain yield. An ample source supply during this phase, either from pre-heading non-structural carbohydrate (NSC) reserves or from post-heading photosynthates, is essential to fill sink sufficiently to obtain rice high yield and good quality (Wei et al., 2018). However, the seed-setting rate and 1000-grain weight showed no synchronous improvement, resulting in no significant yield difference relative to the field, indicating a typical “increased sink but insufficient source” phenomenon. The core reason for stagnant yield is that plant factory conditions prioritize vegetative organ development while restricting panicle and grain formation. Rapid elongation of stems and leaves accompanied by high tissue water content weakens physiological vitality, failing to supply sufficient assimilates for reproductive growth. A short vegetative phase can therefore limit grain yield due to insufficient resource accumulation (Gol et al., 2017). The trade-off between vegetative and reproductive growth, combined with the confounding effects of multiple environmental factors, leads to vigorous vegetative growth yet stagnant grain yield. Further factorial experiments are recommended to clarify the individual impacts of light quality, carbon dioxide concentration and rhizosphere conditions on source-sink translocation and yield formation. To achieve higher yield and desirable grain protein content under optimized cultivation conditions, enhancing sink strength and prolonging nitrogen absorption capacity can be regarded as key breeding objectives (Wingler and Soualiou, 2025).

Environmental differences between the two cultivation systems were closely associated with distinct growth rhythms and phenotypic differentiation in rice. The plant factory provided a relatively stable thermal environment throughout the growth period, accompanied by gradual increases in CO_2_ concentration and relative humidity, whereas field-grown rice experienced a natural seasonal decline in temperature, greater humidity fluctuations, and relatively stable CO_2_ levels (**Figure 2**). Machine-learning-based feature-importance analysis further revealed system-specific patterns among the variables associated with plant height formation. The growth period showed the highest relative importance in both systems, although its contribution was greater in the field than in the plant factory, suggesting that developmental progression remained the dominant factor underlying rice growth dynamics (**Figure 7**). Notably, CO_2_ concentration was more important in the plant factory than in the field, suggesting that CO_2_ dynamics may be closely linked to the accelerated vegetative development observed under controlled-environment conditions. Previous studies have shown that elevated CO_2_ can enhance rice root and aboveground biomass, tiller formation, and photosynthetic capacity, although the magnitude of this response may vary with cultivar type, developmental stage, and nitrogen availability (Jing et al., 2017; Zhou et al., 2021; Hu et al., 2022). However, such CO_2_-induced promotion diminishes as growth progresses, and increased nitrogen supply amplifies the CO_2_ response (Hu et al., 2022). Therefore, the increased importance of CO_2_ observed in this study should be interpreted as a model-derived indication of its potential regulatory relevance rather than direct evidence of causality. These findings suggest that coordinated management of CO_2_ and nitrogen supply, particularly during the transition from vegetative growth to reproductive development, may be a promising strategy to improve source activity, biomass allocation, and yield conversion efficiency in plant-factory rice cultivation. Beyond temperature and CO_2_, light quality (especially red/far-red ratio, R/FR) acts as a critical environmental signal regulating rice tillering and shoot architecture (Huber et al., 2024). A reduced R/FR ratio in the plant factory can promote tiller-bud outgrowth and increase total panicles per unit area, while source-sink imbalance, partly caused by uncoordinated light signals and assimilate allocation, remains the main constraint on yield improvement.As is well known, machine learning excels at extracting statistical relationships and generating quantitative predictions from complex datasets (Noordijk et al., 2024). In our study, all machine learning models exhibited strong predictive performance and good generalizability (**Table 2**), effectively capturing the nonlinear associations between rice growth and environmental variables. These findings provide a reliable theoretical basis and methodological reference for phenotypic prediction in plant factory rice cultivation. Among the five models, XGBoost and GBDT outperformed SVM, RF, and DT in predicting rice plant height across both cultivation systems, achieving high accuracy with R² values above 0.975. Notably, GBDT showed distinct advantages due to its low sensitivity to data skewness and strong anti-overfitting capability (Han et al., 2019; Yoosefzadeh-Najafabadi et al., 2021; Xu et al., 2022), allowing it to better characterize the complex interactions between rice growth and environmental factors. This makes GBDT well-suited for analyzing environmental regulatory effects and for optimizing plant factory cultivation strategies. Admittedly, the current analysis is limited to predicting plant height using a relatively small dataset comprising three cultivars with three biological replicates, which may increase the risk of overfitting and limit the model’s generalizability. Future work will expand the analysis to include traits such as biomass translocation efficiency and grain yield, enabling a more robust investigation of the complex phenotype-environment relationship.

Collectively, this study innovatively selected representative rice cultivars for systematic comparative analysis, integrating phenotypic dissection with machine-learning quantification to supplement and enrich the theoretical foundation for rice cultivation in plant factories. Nevertheless, several limitations still exist, including incomplete monitoring of multiple environmental factors, a limited range of tested cultivars, and a lack of key economic and resource-use indicators, such as energy consumption per unit yield, lighting costs, and water and fertilizer use efficiency. In particular, plant factories impose substantially higher energy demand than conventional food production systems, a critical drawback that is frequently overlooked in relevant research (Orsini et al., 2020; Cai et al., 2025). Therefore, future research should establish a precise, growth-stage-based regulatory system focused on optimizing the integrated management of light, temperature, gas and nutrients during grain filling, so as to reduce operational costs, enhance source strength and sink capacity in a coordinated manner and break the yield bottleneck in rice cultivation in plant factories. Meanwhile, the sustainable development of plant factory systems relies on professional knowledge of botany and agronomy as well as interdisciplinary collaboration with engineering and economics to tackle complex agricultural challenges (Zhuang et al., 2024). Ongoing advances in intelligent environmental regulation and precision cultivation will further unlock the potential of plant factory applications, offering valuable approaches to exploring rice growth plasticity, high-efficiency production and elite germplasm breeding.

## 5 Conclusion

In conclusion, plant factory cultivation markedly shortened rice growth duration and promoted early vegetative development, including tillering, root elongation, total leaf area, and fresh biomass accumulation. It also increased panicle and spikelet numbers, whereas seed-setting rate, 1000-grain weight, panicle dry matter, and final grain yield were not simultaneously improved. Machine-learning-based feature-importance analysis identified growth duration as the dominant predictor of plant height, whereas CO_2_ concentration showed higher relative importance under plant-factory conditions. These findings suggest that controlled-environment cultivation reshapes rice developmental rhythm and vegetative-reproductive allocation, and that improved coordination among source capacity, assimilate transport, and sink demand may be important for converting growth advantages into yield gains. This study provides a physiological basis for understanding rice plasticity and optimizing plant factory-based crop production.

## Data Availability

The raw data supporting the conclusions of this article will be made available by the authors, without undue reservation.
